# Locating Pleistocene Refugia: Comparing Phylogeographic and Ecological Niche Model Predictions

**DOI:** 10.1371/journal.pone.0000563

**Published:** 2007-07-11

**Authors:** Eric Waltari, Robert J. Hijmans, A. Townsend Peterson, Árpád S. Nyári, Susan L. Perkins, Robert P. Guralnick

**Affiliations:** 1 Division of Invertebrate Zoology, American Museum of Natural History, New York, New York, United States of America; 2 International Rice Research Institute, Los Baños, Laguna, Philippines; 3 Natural History Museum & Biodiversity Research Center, University of Kansas, Lawrence, Kansas, United States of America; 4 Department of Ecology and Evolutionary Biology and University of Colorado Museum, University of Colorado, Boulder, Colorado, United States of America; CNRS, France

## Abstract

Ecological niche models (ENMs) provide a means of characterizing the spatial distribution of suitable conditions for species, and have recently been applied to the challenge of locating potential distributional areas at the Last Glacial Maximum (LGM) when unfavorable climate conditions led to range contractions and fragmentation. Here, we compare and contrast ENM-based reconstructions of LGM refugial locations with those resulting from the more traditional molecular genetic and phylogeographic predictions. We examined 20 North American terrestrial vertebrate species from different regions and with different range sizes for which refugia have been identified based on phylogeographic analyses, using ENM tools to make parallel predictions. We then assessed the correspondence between the two approaches based on spatial overlap and areal extent of the predicted refugia. In 14 of the 20 species, the predictions from ENM and predictions based on phylogeographic studies were significantly spatially correlated, suggesting that the two approaches to development of refugial maps are converging on a similar result. Our results confirm that ENM scenario exploration can provide a useful complement to molecular studies, offering a less subjective, spatially explicit hypothesis of past geographic patterns of distribution.

## Introduction

The most compelling evidence that ongoing climate change processes will impact species distributions results is provided by the response of organisms to past climate change [1]. Only 18,000–21,000 years ago, at the Last Glacial Maximum (LGM), landscapes and climates of North America were dramatically different from the present day. Continental ice sheets extended over much of the northern portion of the continent, climatic conditions were considerably drier and colder, and lowered sea levels exposed the Beringian land bridge, connecting the North American and Siberian land masses [2].

LGM distributions of animal and plant species were similarly different from present distributions, particularly in temperate areas, in large part in response to changing climate and landscape conditions [1], [3]. Most species experienced reduction and fragmentation of ranges [4]–[5] because of intrusion by uninhabitable continental ice sheets, distributional shifts and fragmentation of primary habitats such as coniferous forests or deserts, and the development of unfavorable climate conditions beyond species' physiological tolerances. As temperatures warmed from the LGM to present, populations isolated in single or multiple refugia often expanded their geographic distributions as new areas became suitable [6].

Understanding Pleistocene refugial distributions of species has been a core task in historical biogeography for at least four reasons. First, current population genetic structure, within- and between-species genetic diversity, and potential for adaptation to local conditions depend on historical population structure [7]. Second, multiple species in similar habitats may have responded comparably to Pleistocene climate changes [8], thus occupying similar refugial locations. Alternatively, local adaptation to differing regional conditions may have occurred, with distinct paleogeographic implications [e.g. 9]. Third, refugia based on biogeographic evidence can guide paleoenvironmental reconstructions [e.g. 10]. Finally, accurate knowledge of distributional responses to past climate change can provide an excellent calibration for predictions of the consequences of present-day climate change [11]–[12], including the mode and tempo of recolonization of newly available habitats [13].

Pleistocene refugia have been identified based on different types of historical biogeographic evidence. Prior to the 1990s, hypotheses were based primarily on distributions of presumed sister species, disjunctions of species' distributions, fossil distributional data, and paleoenvironmental reconstructions [e.g. 14]. More recently, however, the advent of intraspecific molecular phylogeographic approaches [15] has allowed for stronger inferences about identification of likely refugia based on distributions of genes across landscapes. These phylogeographic studies use patterns of differentiation and similarity to infer locations and disjunctions of past populations, as well as sequences of historical biogeographic isolation events that led to current patterns [16].

Such approaches, however, each have inherent biases and difficulties. For example, use of fossil data alone is problematic because inference of refugia requires precise and correct identification of fossil material during relatively narrow time periods; only in rare cases are taxonomic, spatial, and temporal resolution all sufficient for such inference [17]. Similarly, whereas phylogeographic analyses can locate areas with multiple lineages or high genetic diversity that can be indicative of refugial locations [4], extinctions of genetic variants, incomplete sampling and large-scale range shifts can obscure patterns and make inference of past distributions difficult [18]. Hence, refugial locations are often described with an overly broad geographic brush (e.g. “western United States”; Table S1) in the phylogeographic literature.

Here, we explore a novel method for locating and describing Pleistocene refugia [19]–[20], the use of ecological niche models (ENMs) in conjunction with paleoclimatic reconstructions. ENMs relate known occurrences of species to data describing landscape-level variation in environmental parameters of importance to species' distributional ecology, resulting in models of inferred environmental requirements. These models can be used to predict potential distributional patterns for the species [21]. Such projections assume that a species is in equilibrium with its environmental requirements, i.e., its distribution is mainly determined by the environment, and not by other factors such as competition or dispersal limitation. Similarly, under assumptions of niche conservatism [22], which have been tested extensively [23]–[26], ENMs can be projected onto paleoclimate reconstructions to identify past potential distributions [19], [26]–[27].

One obstacle to applying ENMs for predicting past distributions has been that the spatial resolution of modeled LGM climates was coarse, with grid cells typically 50 km or greater; such coarse-resolution climate data smooth over and obscure sharp environmental gradients and narrow barriers to dispersal. However, here we use recent downscaled high-resolution estimates of LGM climate parameters (see Materials and Methods), permitting a more detailed picture of LGM environments. Marked improvements have also been made in availability of species occurrence data thanks in large part to development of distributed biodiversity data resources [28]–[31]. The combination of these two advances makes possible much greater detail and accuracy in ENM applications to identification of potential Pleistocene refugia.

This suite of ideas has been discussed amply in recent years [25], [32]–[33], but worked examples are only beginning to appear [19]–[20], [26]–[27], [34]–[37]. Studies that have used ENM approaches have focused either on particular regions [e.g. 38], or single-taxon examples [e.g. 39], Peterson and Nyári, submitted]. Here, we test the ability of ENM approaches to reconstruct LGM refugial locations across a diverse suite of 20 species of North American terrestrial vertebrates (Table 1). We chose these species based on the availability of networked occurrence data and detailed phylogeographic predictions for refugia, and we restricted ourselves to North America because of the relatively well-established understanding of its paleoclimate. We find that ENM and phylogeographic predictions are frequently closely correlated, suggesting that the two approaches are converging on similar solutions and that the two in tandem may offer exciting new insights.

**Table 1:** List of the 20 vertebrate taxa examined, number or occurrence points used in ecological niche modeling, and number and source of phylogeographic refugia predicted.

Taxon Name	Number of Occurrences	Number of Refugia	Reference
**Mammals**			
*Arborimus longicaudus*	57	3	69
*Blarina brevicauda*	750	4	70,71
*Dicrostonyx groenlandicus*	89	4	10
*Glaucomys sabrinus*	280	2	54
*Glaucomys volans*	159	1	54
*Lepus arcticus*	34	4	52
*Martes americana*	214	2	72
*Myodes gapperi*	746	3	73
**Amphibians/Reptiles**			
*Ambystoma maculatum*	150	3	74
*Crotalus atrox*	216	4	75
*Desmognathus wrighti*	23	1	76
*Dicamptodon tenebrosus*	40	2	77
*Elaphe obsoleta*	267	3	78
*Eumeces fasciatus*	109	6	79
*Lampropeltis zonata*	39	3	80
*Plethodon idahoensis*	66	1	81
**Birds**			
*Chamaea fasciata*	87	1	82
*Dendragapus obscurus*	174	2	83
*Poecile gambeli*	190	2	84
*Polioptia californica*	38	1	85

## Results

We focused this survey on comparisons of LGM potential distributional summaries between ENM and phylogeographic reconstructions. As such, we were concerned with both the coincidence (measured here as spatial overlap between the two sets of predictions), and the degree to which ENM predictions were broader spatially and less discerning in terms of identifying geographic isolation in LGM refugia (see Materials and Methods). Close concordance between the two approaches would indicate that they are converging on a common solution, which would constitute an improved view of LGM distributional potential in species.

Overlap values of the 20 species examined ranged 0–95% (average 52%); two species showed no overlap between ENM- and phylogeographic-predicted refugia (Table 2). The over-prediction ratio ranged 0–7.9 (average 2.3). In terms of number of refugia predicted, species averaged 2.6 distinct refugia predicted by phylogeographic studies, as compared with 1.5 distinct refugia based on ENM predictions. Comparisons of phylogeographic and ENM predictions for each of the 20 species are available as Figures S1,S2,S3,S4,S5,S6,S7,S8,S9,S10,S11,S12,S13,S14,S15,S16,S17,S18,S19,S20 in Supporting Information; four examples are shown in Figure 1.

**Figure 1:**
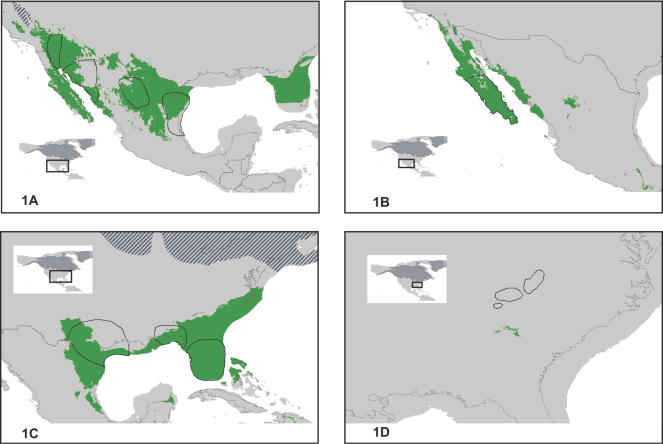
Ecological niche modeling reconstructions of Pleistocene Last Glacial Maximum (LGM) refugia for four taxa. Refugia identified in phylogeographic studies are shown as black outlines. Areas predicted to be refugia are in green, areas not predicted are in gray, and hatching indicates approximate locations of ice sheets [68]. Gray lines indicate present day coastlines. (A) *Crotalus atrox* and (B) *Polioptia californica*, examples of significant overlap and minimal over-prediction. (C) *Elaphe obsoleta*, an example of lack of resolution in ENM predictions in cases of riverine barriers dividing likely LGM refugia. (D) *Desmognathus wrighti*, an example in which both LGM refugium reconstructions are minuscule and in close apposition (although non-overlapping).

**Table 2:** Data comparing ecological niche model predictions and phylogeographic predictions of refugia across 20 vertebrate taxa.

Taxon Name	Overlap (%)	Ratio of ENM predicted pixels to phylogeographic predicted pixels	Number of predicted phylogeographic refugia	Number of corresponding ENM refugia
**Mammals**				
*Arborimus longicaudus*	61.6	1.02	3	1
*Blarina brevicauda*	20.4	1.05	4	3
*Dicrostonyx groenlandicus*	67.7	1.38	4	3
*Glaucomys sabrinus*	49.9	2.18	2	2
*Glaucomys volans*	59.1	0.99	1	1
*Lepus arcticus*	10.6	3.09	4	2
*Martes americana*	46.0	2.87	2	2
*Myodes gapperi*	80.5	2.69	3	2
**Amphibians/Reptiles**				
*Ambystoma maculatum*	41.7	2.05	3	1
*Crotalus atrox*	69.2	2.69	4	3
*Desmognathus wrighti*	0.0	0.08	1	0
*Dicamptodon tenebrosus*	79.3	7.90	2	1
*Elaphe obsoleta*	71.0	1.70	3	1
*Eumeces fasciatus*	43.0	1.04	6	2
*Lampropeltis zonata*	65.9	1.27	3	1
*Plethodon idahoensis*	0.0	0.00	1	0
**Birds**				
*Chamaea fasciata*	65.9	2.69	1	1
*Dendragapus obscurus*	79.7	5.05	2	1
*Poecile gambeli*	39.2	4.52	2	2
*Polioptia californica*	94.7	2.63	1	1
**Average**	**52.3**	**2.34**	**30/52**	

Results of the spatially-corrected correlations between ENM-predicted suitable habitat at the LGM and phylogeographically predicted refugial locations are shown in Table 3. As expected, using a spatial correlation approach dramatically lowered the degrees of freedom and thus the power of the statistical test to distinguish between random and nonrandom correlations. However, even so, 14 of the 20 comparisons showed a significantly stronger than random correlation between the two different predictions (e.g. Figure 1A,B). In three of the six cases where we could not reject the null hypothesis of no association, the *P*-values missed the set significance criterion of 0.05 only marginally (i.e. 0.05<*P*<0.10).

**Table 3:** Spatially-corrected correlations between ecological niche model predictions and phylogeographic predictions of refugial locations at the Last Glacial Maximum across 20 vertebrate taxa.

Taxon Name	Overlap (%)	Number of pixels in grid (uncorrected d.f.)	Pearson's *r*	Corrected d.f.	Corrected F	Corrected *P*
**Mammals**						
*Arborimus longicaudus*	61.6	2074	0.483	566.2	172.1	<0.001**
*Blarina brevicauda*	20.4	2366	0.154	128.6	3.15	0.078
*Dicrostonyx groenlandicus*	67.7	2989	0.496	17.73	5.79	0.027*
*Glaucomys sabrinus*	49.9	2781	0.261	58.2	4.26	0.043*
*Glaucomys volans*	59.1	1791	0.570	46.9	22.7	<0.001**
*Lepus arcticus*	10.6	2329	0.009	614.8	0.054	0.815
*Martes americana*	46.0	2322	0.193	77.9	3.02	0.086
*Myodes gapperi*	80.5	1742	0.431	88.7	20.27	<.001**
**Amphibians/Reptiles**						
*Ambystoma maculatum*	41.7	1809	0.274	118.5	9.64	0.002*
*Crotalus atrox*	69.2	1234	0.342	114.8	15.24	<.001**
*Desmognathus wrighti*	0.0	n/a	n/a	n/a	n/a	n.s.
*Dicamptodon tenebrosus*	79.3	2217	0.285	868.9	77.1	<.001**
*Elaphe obsoleta*	71.0	1819	0.511	83.9	29.61	<.001**
*Eumeces fasciatus*	43.0	1809	0.388	107.1	18.94	<.001**
*Lampropeltis zonata*	65.9	2096	0.586	344.3	180.3	<.001**
*Plethodon idahoensis*	0.0	n/a	n/a	n/a	n/a	n.s.
**Birds**						
*Chamaea fasciata*	65.9	1735	0.425	446.9	98.5	<.001**
*Dendragapus obscurus*	79.7	2322	0.322	91.6	10.59	0.001**
*Poecile gambeli*	39.2	2352	0.154	157.3	3.83	0.052
*Polioptia californica*	94.7	1323	0.569	307.7	147.4	<.001**

* indicates significance at less than 0.05 *P*-value.

** indicates significance at less than 0.001 *P*-value.

‘n.s.’ indicates non-significance.

## Discussion

Advances in molecular methods and incorporation of novel analytical techniques (e.g. coalescent approaches; [40]) have produced a flood of literature examining phylogeographic patterns for many species, often identifying areas that constituted past refugia (see [5] for one review). Given the time frame of resolution of coalescent methods, many of these studies focus on Pleistocene refugia. The phylogeographic approaches, however, have two important limitations that are directly relevant to this study. First, the geography of the lineages and their splitting events is reconstructed subjectively via reference to inferred paleodistributional shifts rather than incorporating geography and paleoenvironmental conditions explicitly. Second, dating key events precisely from molecular data presents numerous challenges [41]. Here, we provide the first broad survey of taxa to evaluate how well ‘back-casting’ of ecological niche models can complement phylogeographic approaches in identifying refugia. A clear advantage of the ENM approaches is that they provide an explicit tie to environment and geography not available from the phylogeographic analyses.

The question posed in this paper was simple: do ENM and phylogeographic techniques, which utilize radically different data sources and analytical approaches, lead to concordant reconstructions of LGM biogeography of species? Overall, we found that 14 of 20 species examined had significant agreement between the two reconstructions (Table 3; Figure 1), although differences do exist. These results, along with considerable work published elsewhere and by numerous research groups [21], [42]–[46], support the idea that the bioclimatic variables used in our ENM predictions (see Materials and Methods) are of importance to the past and present distribution of the species analyzed [21] and may be suitable for other species. In the paragraphs that follow, we discuss patterns of concordance, as well as reasons for the differences. We also attempt to take initial steps toward a synthetic methodology for incorporating paleodistributional reconstructions in systematic studies.

### Factors reducing overlap of LGM reconstructions

Multiple factors, both extrinsic and intrinsic, can reduce overlap between ENM and phylogeographic LGM refugial reconstructions. First, we note that decisions involved in combining results from different ENM algorithms and paleoclimatic reconstructions is a complex task [45], [47]. Because our approach focused on increasing resolution and definition of refugia, we may underestimate LGM distributional potential somewhat. Considerations regarding threshold values can act similarly to yield broader or narrower areas predicted habitable [46], with the same costs and benefits. All of these factors should not be overlooked when assembling ENM predictions.

Differences between ENM and phylogeographic results may also spring from the effects of biotic interactions on species' distributional potential. Considerable debate exists regarding the degree to which niche models capture these interaction effects and the degree to which interaction effects may disrupt predictivity over space and time [21]–[22], [48]–[49]. For example, dispersal limitations constrain species from colonizing the full spatial extent of their potential distributional areas (e.g., the potential distributional areas around Newfoundland for *Dendragapus obscurus*, Blue Grouse, of western North America). The reptiles, small mammals, and non-migratory birds in the study could be expected to have roughly similar dispersal capabilities. In contrast, both of the species showing null overlap were salamanders, which generally have lower dispersal rates [50], although the average overlap among all four salamander species was not significantly lower than in other taxa. Evolutionary history may also be a constraint, whereby potential distributional areas are not inhabited owing to presence of a sister taxon instead of the species in question [24]. This appears to be the case for *Myodes* (*Clethrionomys*) *gapperi* (Southern Red-backed Vole), where the closely related *M. rutilus* likely occupied the Beringian LGM refugium identified in our analyses [51].

Another consideration is that certain biogeographic barriers are more easily detectable in ENM analyses than others, given the nature of the paleoclimatic layers currently available. Mountain ranges or large ice sheets are reflected in climate layers, as they present major differences in temperature and precipitation profiles. Smaller barriers, however, may be less easy to detect, particularly river systems, which are not generally represented in climatic data sets. Such small but strongly vicariant features have been implicated in separating populations in groups such as *Blarina brevicauda* (Northern Short-tailed Shrew), *Eumeces fasciatus* (Five-lined Skink), and *Elaphe obsoleta* (Eastern Ratsnake; Figure 1C), and may explain some discordance in results between ENM and phylogeographic prediction. Overall, however, our results indicate that the ENM approach is quite powerful in estimating LGM distributions.

We had two cases of null overlap between the two refugial predictions (*Desmognathus wrighti* (Pygmy Salamander) and *Plethodon idahoensis* (Coeur d'Alene Salamander). Both have very small geographic distributions, although LGM ranges were reconstructed successfully for other species with small ranges (e.g. *Arborimus longicaudus*; Red Tree Vole). These two ‘failures’ in the ENM approach, may be instructive, as they are quite different. In the case of *D. wrighti*, the ENM LGM refugial predictions are shifted just 100 km south of the phylogeographic reconstructions (Figure 1D). This case may be one in which the spatially-explicit ENM predictions provide a qualitative advantage over the phylogeographic approaches by identifying refugial areas much more precisely. In *P. idahoensis*, however, no suitable conditions were identified near the species' present distribution, and discordance about suitable habitats was found between modeling algorithms, suggesting that the ENM predictions in this case may be of poor quality owing to incomplete representation of environments in the training data sets. A related problem is that incomplete knowledge of past landscapes and environments may lead to erroneous conclusions. For example, the *Lepus arcticus* (Arctic Hare) phylogeographic prediction [52] is heavily dependent on the northern limits of ice sheets in North America, which are still not well resolved [53]. The result that two of the four ENM-predicted refugia for *L. arcticus* are in areas considered to be ice-covered at LGM may actually be correct, but will have to await additional and improved information on LGM landscapes and environments.

Our approach to model consilience was designed to minimize over-prediction of refugial distributional areas. As a result, this approach not only had lower over-prediction ratios (Table 2), but also was more successful in identifying LGM barriers to dispersal and gene flow between refugia than less conservative approaches explored initially (not shown). Whether some level of over-prediction still remains, however, is a question that will await further analysis and testing. Of 15 species with multiple phylogeography-based refugia, ENM-based approaches discovered multiple corresponding refugia in nine species, and yet others showed range constrictions and fragmentation that may have contributed to isolation of populations at LGM. In the cases in which ENMs did not find distinct refugia, factors discussed above causing reduced overlap may be playing a role, especially small barriers such as rivers. In addition, discrepancy in corresponding refugia may in fact be due to the wide range of phylogeographic refugia predictions, some of which are very coarse (e.g. *Glaucomys sabrinus*; Northern Flying Squirrel). Because ENM approaches in such cases are often more refined, the use of ENM to complement phylogeographic predictions will likely improve inference of Pleistocene refugia (see below).

### Improving LGM refugial reconstructions

ENM methods are only beginning to be applied to the challenge of reconstructing paleodistributions [19]–[20], [38] and others mentioned in introduction], but we believe this approach to have great potential. New, higher-resolution paleoenvironmental data sets such as the LGM climate data used herein are increasingly available, so further related research in this realm should be increasingly fruitful. Finer-scale resolution, and additional, biologically-relevant paleoenvironmental layers (e.g., of soil types, hydrology, land cover, etc.) will likely increase further the quality and resolution of ENM predictions.

A related issue is that, for the moment at least, our ENM refugial projections are solely to LGM conditions, and not to earlier or later conditions. Each lineage, obviously, has a history that extends over previous Pleistocene glaciation events, and back into the Pliocene or earlier. For some of the species examined, phylogeographic predictions extend even further back into history [e.g. 54]. We envision that in the near future, deeper-history reconstructions will become available, which should provide a picture of climatic conditions across the alternating warm and cold periods during the Pleistocene [55]. Including these additional time slices representing other points in the Pleistocene and early Holocene [56] will allow detailed examination of changes in paleodistributions of species, and thus be greatly useful in historical biogeographic studies.

Phylogeographic analyses are expensive, in terms of both time and resources. ENM approaches offer a first approximation of the spatial distribution and extent of potential Pleistocene refugia, an approximation that will likely improve as more environmental reconstructions become available. It is tempting to ask the question of which of the two methods explored here (ENM and phylogeography) is better in reconstructing Pleistocene refugia. Of course, each has advantages and disadvantages that are only beginning to be appreciated thanks to the novelty of the ENM approaches. We do not see the two approaches as competing; rather, the rigorous, population genetic nature of the phylogeographic approaches is made more explicit spatially and temporally by the ENM approaches, making for an even more quantitative product.

We recommend that biogeographers consider these methods both for experimental design and for comparison with phylogeographic results. For example, ENM predictions can be used in study design to pick key regions for sampling, corresponding to potential LGM refugial isolates. Later, tandem implementation of ENM and phylogeographic techniques will produce a better understanding of species' distributions in the Pleistocene. Of particular interest are cases in which phylogeographic and ENM approaches do not overlap, as confidence in one set of results or the other is called into question, providing some level of falsifiability of reconstructions. Finally, ENM methods can incorporate phylogenetic lineage-specific ENMs, applications of which are now beginning to appear [38]; Peterson and Nyári, submitted]. Similarly, we note that the *geography* of paleogeographic reconstructions in phylogeographic studies has been subjective, so we hope that this pairing of methods can lend increased rigor to that field. Such methods, especially when combined with primary phylogeographic and fossil data will also bring an exciting new dimension to biogeographic research.

## Materials and Methods

### Environmental data

To create LGM climate layers for use in the ENMs, we used current and LGM monthly climate data at 2.5′ spatial resolution. Current climate data from the WorldClim database [57] were used, whereas LGM climate data were drawn from general circulation model (GCM) simulations from two models: the Community Climate System Model (CCSM) [58] and the Model for Interdisciplinary Research on Climate (MIROC, version 3.2) [59]. The original GCM data were downloaded from the PMIP2 website (http://www.pmip2.cnrs-gif.fr), with a spatial resolution of 2.8°, or roughly 300×300 km.

We created monthly climate surfaces at 2.5′ spatial resolution as follows. First, at the native coarse resolutions, we calculated the differences between LGM and recent (pre-industrial) conditions. These differences were then interpolated to 2.5′ resolution using the spline function in ArcInfo (ESRI, Redlands, CA) with the tension option. Finally, the interpolated difference maps were added to the WorldClim current climate data. This procedure had the dual advantage of producing data at a resolution relevant to the spatial scale of analysis, and of calibrating the downscaled LGM climate data to actual observed climate conditions.

ENMs were based on the 19 bioclimatic variables in the WorldClim data set [57]. These variables represent summaries of means and variation in temperature and precipitation, and likely summarize dimensions of climate particularly relevant in determining species distributions (Text S1 in Supporting Information). All ENM development (i.e., present-day analyses) were developed within one of three sub-regions: ‘North,’ 30–180°W, 30–85°N; ‘West,’ 100–130°W, 20–60°N; ‘East/South,’ 50–130°W, 5–55°N.

### Species selection and occurrence data

We focused on North American terrestrial vertebrates for three reasons: (1) ample individual phylogeographic analyses have been developed; (2) occurrence data are abundant and are already networked for ENM development; and (3) LGM environments of the continent are relatively well understood [1], [3]. We focused on taxa for which detailed phylogeographic studies (i.e., covering the entire range of the species) are available, and attempted to include a diversity of range sizes (narrow to broad), choosing 8 mammals, 4 reptiles, 4 amphibians, and 4 non-migratory birds (Table 1). Occurrence data for the 20 taxa is listed in Dataset S1 in Supporting Information.

We used networked biodiversity information systems of natural history collection data (e.g. MaNIS, HerpNET, ORNIS and other DiGIR providers) to collate species occurrence information from multiple repositories for the focal taxa, thus drawing data from numerous institutions (see Text S2 in Supporting Information). We first removed duplicate records for the same species collected at the same site, and then assigned geographic coordinates based on textual locality descriptions (localities within 0.1° of one another were removed to reduce effects of spatial autocorrelation) using a combination of the Biogeomancer workbench [60] and the GeoLocate desktop program. To avoid basing ENMs on imprecise occurrence data, only records with geographic uncertainty [61] of less than 15 km were retained for analysis.

### ENM approaches

Recent studies have advised a consensus approach in ENM development, in which multiple algorithms are used [46], [62]. Thus, we applied both the Maxent [63] and GARP [64] algorithms to construct ENMs. Both programs generate ENMs using only presence records, contrasting them with pseudo-absence data sampled from the remainder of the study area. In each case, we developed present-day ENMs based on occurrences within the mask appropriate to the particular species, but then projected the ENM to both present-day and LGM conditions across all of North America. We chose not to mask LGM ice sheets because their margins are still under debate [53], and including these likely unsuitable areas results in a more conservative approach and avoids additional assumption making.

For Maxent (version 2.3) [63], we used the default convergence threshold (10^−5^) and maximum number of iterations (500) values, using 25% of localities for model training. We let the program select both suitable regularization values and functions of environmental variables automatically, which it achieves based on considerations of sample size. Maxent outputs a continuous probability value, ranging from 0 to 100, an indicator of relative suitability for the species, based on the principle of maximum entropy, as constrained by the input occurrence data.

We also used Desktop GARP (version 1.1.6) [64] to construct ENMs. For each species, we created 100 random replicate GARP models, using the default parameters of convergence limit (0.01) and maximum iterations (1000). To select the best ENMs from among the replicate model runs, we followed Anderson et al. [43] in prioritizing low-omission models for further consideration (the 20% of replicate models showing lowest extrinsic omission error), and then retaining the central 50% of the distribution of areas predicted present to avoid models showing high commission error rates.

### Thresholds and model combination

**Figure 2:**
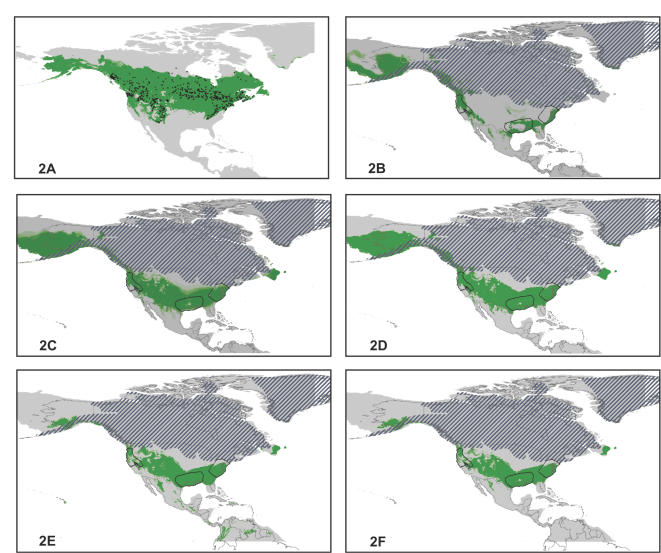
Process diagram summarizing the assembly of ecological niche model predictions for Pleistocene distributions. For continuous predictions, colors shift from gray to green as prediction values increase from 0 to 10 (GARP) or 100 (Maxent). For binary predictions, areas predicted as suitable at Last Glacial Maximum are shown in green, and those not so predicted are in gray. Hatching indicates approximate locations of ice sheets [68], and dotted lines indicate present day coastlines. (A) Present day occurrences (black dots) and binary ENM prediction of *Myodes gapperi* using GARP, based on a threshold of 5 of 10 replicate models. (B) LGM projection of present-day ENM to climates reconstructed in CCSM model for *M. gapperi* using GARP. (C) LGM projection of present-day ENM to climates reconstructed in MIROC model for *M. gapperi* using GARP. (D) LGM binary prediction of *M. gapperi* from CCSM or MIROC models, using GARP threshold of 5. (E) LGM binary prediction of *M. gapperi* from CCSM or MIROC models, using Maxent threshold of 10. (F) Logical combination of GARP5 ‘and’ Maxent10 models for LGM prediction of *M. gapperi*.

Given two LGM climate reconstructions and two ENM algorithms, we have four LGM reconstructions of suitable conditions for each species (Figure 2). Although the individual model projections and similarities and differences among them are relevant and interesting, for the purposes of the present analysis we opted to seek consensus among different LGM projections for each species. Hence, to reconcile results, we first chose thresholds for GARP (raw output ranged as integers 0–10) and Maxent (raw output ranged as a real number 0–100) results. For GARP, we used a threshold of >5 (G5), or that area predicted present by at least half of the final 10 replicate models [20]. For Maxent, we used a value of 10 (M10), which has also been suggested as an appropriate threshold [46]. In all cases, these thresholds identified smaller areas than a lowest presence threshold that yielded zero omission error, thus resulting in more restricted pictures of potential LGM distributions. Application of these thresholds effectively rendered each LGM projection into a binary form, predicting either potential presence or absence across North America.

Next, we generated a final consensus model for each species. An initial approach considered any area that any of the models deemed suitable as representing an area of potential distribution (equivalent to an “or” operation in combining layers). However, exploration of these results showed relatively broad areas predicted as suitable, suggesting that a more conservative definition of suitable habitat was desirable. Our conservative approach for generating a model consensus was to keep any area predicted by either climate model but to discard areas not predicted as suitable by both algorithms (equivalent to an “or” operation for climate models and an “and” operation for algorithms).

### Comparing ENM-predicted and phylogeographic refugia hypotheses

Prior to and independent of calculating ENM predictions, we converted textual or map-based descriptions of refugial locations from the phylogeographic literature (Table 1) into a geographic footprint. In the case of maps, we transcribed their locations as polygons directly into vector shapefiles in ArcGIS. When only textual descriptions of refugial locations were available, we converted descriptions into polygon footprints using techniques similar to those for georeferencing occurrence localities [60]. Of course, some refugial locations are more precise than others, and this is reflected in the polygon-based summaries used here: for example, “Queen Charlotte Islands” is relatively precise, whereas “Central Highlands west of the Mississippi River” is relatively imprecise.

Then, we compared quantitatively amounts of areal overlap between phylogeographic- and ENM-predicted footprints via three quantitative measures of overlap for each species examined. The first measure is the percentage of phylogeographic-predicted area also predicted by ENM [65, equation 2]. The second measure (over-prediction ratio, related to equation 1 in Hijmans and Graham [65]) is the ratio of the area of ENM-suitable habitat to the phylogeographic-predicted area; we assume that over-prediction ratios much larger than unity represent poorer LGM distributional reconstructions. Third, we compared ENM and phylogeographic predictions in terms of the number of distinct predicted refugia (i.e., disjunct polygons).

### Significance testing

To test whether coincidence between the two predictions of refugial locations was better than random, we analyzed spatial correlations using the method of Dutilleul [66], as implemented in Spatial Analysis in Macroecology (SAM) [67]. This method adjusts the degrees of freedom in a correlation analysis based on measures of spatial autocorrelation in both datasets. This adjustment is necessary because we expect spatial autocorrelation *a priori* given strong environmental gradients running both north-south (latitudinal) and east to west (North American mountain ranges). To assure that the test could be computed in a reasonable amount of time, we aggregated the model outputs by a factor of 15–30, cut the grids to those latitudinal areas that broadly contained predicted suitable areas, and then outputted an *xyz* grid of longitude, latitude, and either suitable or unsuitable for both ENM and phylogeographic predictions.

## Supporting Information

Dataset S1 Occurrence data/coordinates (degrees Latitude, Longitude) of the 20 species examined.
(0.27 MB XLS)

Table S1 Predicted phylogeographic refugia of the 20 taxa examined.
(0.07 MB DOC)

Text S1 List of 19 environmental variables from the WorldClim database [57] used in ecological niche modeling.
(0.02 MB DOC)

Text S2 List of data providers from which biodiversity occurrence data were obtained.
(0.03 MB DOC)

Figure S1 Ecological niche modeling reconstructions of Pleistocene Last Glacial Maximum (LGM) refugia for *Arborimus longicaudus*. Refugia identified in phylogeographic studies are shown as black outlines. Areas predicted to be refugia are in green, areas not predicted are in gray, and hatching indicates approximate locations of ice sheets [68]. Dotted lines indicate present day coastlines.
(3.53 MB EPS)

Figure S2 Ecological niche modeling reconstructions of Pleistocene Last Glacial Maximum (LGM) refugia for *Blarina brevicauda*. Refugia identified in phylogeographic studies are shown as black outlines. Areas predicted to be refugia are in green, areas not predicted are in gray, and hatching indicates approximate locations of ice sheets [68]. Dotted lines indicate present day coastlines.
(5.43 MB EPS)

Figure S3 Ecological niche modeling reconstructions of Pleistocene Last Glacial Maximum (LGM) refugia for *Dicrostonyx groenlandicus*. Refugia identified in phylogeographic studies are shown as black outlines. Areas predicted to be refugia are in green, areas not predicted are in gray, and hatching indicates approximate locations of ice sheets [68]. Dotted lines indicate present day coastlines.
(5.54 MB EPS)

Figure S4 Ecological niche modeling reconstructions of Pleistocene Last Glacial Maximum (LGM) refugia for *Glaucomys sabrinus*. Refugia identified in phylogeographic studies are shown as black outlines. Areas predicted to be refugia are in green, areas not predicted are in gray, and hatching indicates approximate locations of ice sheets [68]. Dotted lines indicate present day coastlines.
(6.39 MB EPS)

Figure S5 Ecological niche modeling reconstructions of Pleistocene Last Glacial Maximum (LGM) refugia for *Glaucomys volans*. Refugia identified in phylogeographic studies are shown as black outlines. Areas predicted to be refugia are in green, areas not predicted are in gray, and hatching indicates approximate locations of ice sheets [68]. Dotted lines indicate present day coastlines.
(3.74 MB EPS)

Figure S6 Ecological niche modeling reconstructions of Pleistocene Last Glacial Maximum (LGM) refugia for *Lepus arcticus*. Refugia identified in phylogeographic studies are shown as black outlines. Areas predicted to be refugia are in green, areas not predicted are in gray, and hatching indicates approximate locations of ice sheets [68]. Dotted lines indicate present day coastlines.
(3.84 MB EPS)

Figure S7 Ecological niche modeling reconstructions of Pleistocene Last Glacial Maximum (LGM) refugia for *Martes americana*. Refugia identified in phylogeographic studies are shown as black outlines. Areas predicted to be refugia are in green, areas not predicted are in gray, and hatching indicates approximate locations of ice sheets [68]. Dotted lines indicate present day coastlines.
(6.17 MB EPS)

Figure S8 Ecological niche modeling reconstructions of Pleistocene Last Glacial Maximum (LGM) refugia for *Myodes gapperi*. Refugia identified in phylogeographic studies are shown as black outlines. Areas predicted to be refugia are in green, areas not predicted are in gray, and hatching indicates approximate locations of ice sheets [68]. Dotted lines indicate present day coastlines.
(5.79 MB EPS)

Figure S9 Ecological niche modeling reconstructions of Pleistocene Last Glacial Maximum (LGM) refugia for *Ambystoma maculatum*. Refugia identified in phylogeographic studies are shown as black outlines. Areas predicted to be refugia are in green, areas not predicted are in gray, and hatching indicates approximate locations of ice sheets [68]. Dotted lines indicate present day coastlines.
(4.24 MB EPS)

Figure S10 Ecological niche modeling reconstructions of Pleistocene Last Glacial Maximum (LGM) refugia for *Crotalus atrox*. Refugia identified in phylogeographic studies are shown as black outlines. Areas predicted to be refugia are in green, areas not predicted are in gray, and hatching indicates approximate locations of ice sheets [68]. Dotted lines indicate present day coastlines.
(3.06 MB EPS)

Figure S11 Ecological niche modeling reconstructions of Pleistocene Last Glacial Maximum (LGM) refugia for *Desmognathus wrighti*. Refugia identified in phylogeographic studies are shown as black outlines. Areas predicted to be refugia are in green, areas not predicted are in gray, and hatching indicates approximate locations of ice sheets [68]. Dotted lines indicate present day coastlines.
(1.67 MB EPS)

Figure S12 Ecological niche modeling reconstructions of Pleistocene Last Glacial Maximum (LGM) refugia for *Dicamptodon tenebrosus*. Refugia identified in phylogeographic studies are shown as black outlines. Areas predicted to be refugia are in green, areas not predicted are in gray, and hatching indicates approximate locations of ice sheets [68]. Dotted lines indicate present day coastlines.
(3.37 MB EPS)

Figure S13 Ecological niche modeling reconstructions of Pleistocene Last Glacial Maximum (LGM) refugia for *Elaphe obsoleta*. Refugia identified in phylogeographic studies are shown as black outlines. Areas predicted to be refugia are in green, areas not predicted are in gray, and hatching indicates approximate locations of ice sheets [68]. Dotted lines indicate present day coastlines.
(3.67 MB EPS)

Figure S14 Ecological niche modeling reconstructions of Pleistocene Last Glacial Maximum (LGM) refugia for *Eumeces fasciatus*. Refugia identified in phylogeographic studies are shown as black outlines. Areas predicted to be refugia are in green, areas not predicted are in gray, and hatching indicates approximate locations of ice sheets [68]. Dotted lines indicate present day coastlines.
(3.94 MB EPS)

Figure S15 Ecological niche modeling reconstructions of Pleistocene Last Glacial Maximum (LGM) refugia for *Lampropeltis zonata*. Refugia identified in phylogeographic studies are shown as black outlines. Areas predicted to be refugia are in green, areas not predicted are in gray, and hatching indicates approximate locations of ice sheets [68]. Dotted lines indicate present day coastlines.
(2.69 MB EPS)

Figure S16 Ecological niche modeling reconstructions of Pleistocene Last Glacial Maximum (LGM) refugia for *Plethodon idahoensis*. Refugia identified in phylogeographic studies are shown as black outlines. Areas predicted to be refugia are in green, areas not predicted are in gray, and hatching indicates approximate locations of ice sheets [68]. Dotted lines indicate present day coastlines.
(6.04 MB EPS)

Figure S17 Ecological niche modeling reconstructions of Pleistocene Last Glacial Maximum (LGM) refugia for *Chamaea fasciata*. Refugia identified in phylogeographic studies are shown as black outlines. Areas predicted to be refugia are in green, areas not predicted are in gray, and hatching indicates approximate locations of ice sheets [68]. Dotted lines indicate present day coastlines.
(2.85 MB EPS)

Figure S18 Ecological niche modeling reconstructions of Pleistocene Last Glacial Maximum (LGM) refugia for *Dendragapus obscurus*. Refugia identified in phylogeographic studies are shown as black outlines. Areas predicted to be refugia are in green, areas not predicted are in gray, and hatching indicates approximate locations of ice sheets [68]. Dotted lines indicate present day coastlines.
(4.39 MB EPS)

Figure S19 Ecological niche modeling reconstructions of Pleistocene Last Glacial Maximum (LGM) refugia for *Poecile gambeli*. Refugia identified in phylogeographic studies are shown as black outlines. Areas predicted to be refugia are in green, areas not predicted are in gray, and hatching indicates approximate locations of ice sheets [68]. Dotted lines indicate present day coastlines.
(5.56 MB EPS)

Figure S20 Ecological niche modeling reconstructions of Pleistocene Last Glacial Maximum (LGM) refugia for *Polioptia californica*. Refugia identified in phylogeographic studies are shown as black outlines. Areas predicted to be refugia are in green, areas not predicted are in gray, and hatching indicates approximate locations of ice sheets [68]. Dotted lines indicate present day coastlines.
(2.12 MB EPS)
