# Supplementary material for: Locating Pleistocene Refugia: Comparing Phylogeographic and Ecological Niche Model Predictions
Source: PLoS One. 2007 Jul 11;2(7):e563. doi: 10.1371/journal.pone.0000563 (PMC1905943; doi:10.1371/journal.pone.0000563)
Supplement: Table S1 — Predicted phylogeographic refugia of the 20 taxa examined. (0.07 MB DOC) [file pone.0000563.s002.doc]

Table S1. List of the 20 vertebrate taxa examined, with the phylogeographic refugia predicted.

| **Taxon** | **Refugium 1** | **Refugium 2** | **Refugium 3** | **Refugium 4** | **Refugium 5** | **Refugium 6** |
| --- | --- | --- | --- | --- | --- | --- |
| **Mammals** |  |  |  |  |  |  |
| *Arborimus longicaudus* | N Oregon Coast Range | N Oregon Cascades | Siskiyou Mountains |  |  |  |
| *Blarina brevicauda* | Appalachians | Oklahoma, Texas W of Mississippi R | Coastal plain W of Mississippi R | Coastal Plain E of Mississippi R |  |  |
| *Dicrostonyx groenlandicus* | Beringia | High Canadian Arctic Refugium 1 | High Canadian Arctic Refugium 2 | High Canadian Arctic Refugium 3 |  |  |
| *Glaucomys sabrinus* | Pacific Northwest | Southeast US |  |  |  |  |
| *Glaucomys volans* | Southeast US + Mexico |  |  |  |  |  |
| *Lepus arcticus* | High Canadian Arctic Refugium 1 | High Canadian Arctic Refugium 2 | High Canadian Arctic Refugium 3 | Atlantic Coast of Baffin Island |  |  |
| *Martes americana* | Western US | Eastern US |  |  |  |  |
| *Myodes gapperi* | Western US | Central US | Eastern US |  |  |  |
| **Amphibians/Reptiles** |  |  |  |  |  |  |
| *Ambystoma maculatum* | Southeast coastal | Southern Appalachians | Eastern interior |  |  |  |
| *Crotalus atrox* | Central-south Sonoran desert | California-Arizona Border | Mapimian region - Chihauhuan Desert | Gulf Coast/ Tamaulipan Plain |  |  |
| *Desmognathus wrighti* | Appalachian Refugium |  |  |  |  |  |
| *Dicamptodon tenebrosus* | Columbia River Valley | Klamath/Siskiyou Mountains |  |  |  |  |
| *Elaphe obsoleta* | Texas | Florida W of Apalachicola R | Southern Florida E of Apalachicola R |  |  |  |
| *Eumeces fasciatus* | Northern Texas | Central Highlands W of Mississippi R | Coastal Plain E of Mississippi R | Appalachians | Atlantic Coastal Plain | Driftless Zone |
| *Lampropeltis zonata* | Southern California | Central coastal California | N California to Columbia R |  |  |  |
| *Plethodon idahoensis* | Clearwater drainage, Idaho |  |  |  |  |  |
| **Birds** |  |  |  |  |  |  |
| *Chamaea fasciata* | Southern California |  |  |  |  |  |
| *Dendragapus obscurus* | Western portion of current range | Eastern portion of current range |  |  |  |  |
| *Poecile gambeli* | Southern Sierra Nevada | Southern Rocky Mountains |  |  |  |  |
| *Polioptia californica* | Southern Baja California |  |  |  |  |  |
