# Supplementary material for: Locating Pleistocene Refugia: Comparing Phylogeographic and Ecological Niche Model Predictions
Source: PLoS One. 2007 Jul 11;2(7):e563. doi: 10.1371/journal.pone.0000563 (PMC1905943; doi:10.1371/journal.pone.0000563)
Supplement: Text S1 — List of 19 environmental variables from the WorldClim database [57] used in ecological niche modeling. (0.02 MB DOC) [file pone.0000563.s003.doc]

Text S1. List of 19 environmental variables from the WorldClim database [57] used in ecological niche modeling.

BIO1 = Annual Mean Temperature
BIO2 = Mean Diurnal Range (Mean of monthly (max temp - min temp))
BIO3 = Isothermality (* 100)
BIO4 = Temperature Seasonality (standard deviation *100)
BIO5 = Max Temperature of Warmest Month
BIO6 = Min Temperature of Coldest Month
BIO7 = Temperature Annual Range
BIO8 = Mean Temperature of Wettest Quarter 
BIO9 = Mean Temperature of Driest Quarter
BIO10 = Mean Temperature of Warmest Quarter
BIO11 = Mean Temperature of Coldest Quarter
BIO12 = Annual Precipitation
BIO13 = Precipitation of Wettest Month
BIO14 = Precipitation of Driest Month
BIO15 = Precipitation Seasonality (Coefficient of Variation)
BIO16 = Precipitation of Wettest Quarter
BIO17 = Precipitation of Driest Quarter
BIO18 = Precipitation of Warmest Quarter
BIO19 = Precipitation of Coldest Quarter
