# Supplementary material for: Locating Pleistocene Refugia: Comparing Phylogeographic and Ecological Niche Model Predictions
Source: PLoS One. 2007 Jul 11;2(7):e563. doi: 10.1371/journal.pone.0000563 (PMC1905943; doi:10.1371/journal.pone.0000563)
Supplement: Text S2 — List of data providers from which biodiversity occurrence data were obtained. (0.03 MB DOC) [file pone.0000563.s004.doc]

Text S2. List of data providers from which biodiversity occurrence data were obtained.

Bernice P. Bishop Museum

Biodiversity Research Center at the University of Kansas

California Academy of Sciences

Colección de Mamíferos de la Sierra Volcánica Transversal de México

Cornell University Museum of Vertebrates

Department of Zoology, Arizona State University

Field Museum of Natural History

Florida Museum of Natural History

Illinois Natural History Survey

Instituto de Biologia Universidad Nacional Autónoma de México

Los Angeles County Museum, Museum of Vertebrate Zoology

Louisiana State University Museum of Natural Science

Michigan State University Museum

Monte L. Bean Museum, Brigham Young University

Museum of Comparative Zoology, Harvard University

Museum of Southwestern Biology

Museum of Texas Tech University

Royal Ontario Museum

San Diego Natural History Museum

Santa Barbara Museum of Natural History

Slater Museum of Natural History, University of Puget Sound

Sternberg Museum of Natural History, Fort Hayes

Swedish Museum of Natural History

Texas Cooperative Wildlife Collection, Texas A & M University

The Burke Museum of Natural History and Culture

UNAM-MZFC Herpetologia

University of Alaska Museum of the North

University of Alberta Museum of Zoology

University of California, Davis Association of Biological Collections

University of Colorado Museum of Natural History

University of Illinois Museum of Natural History

University of Michigan Museum of Zoology

University of Minnesota James F. Bell Museum of Natural History

University of Nebraska State Museum

University of Texas at El Paso Museum

University of Texas-Arlington

University of Texas-Austin

Zoological Museum, University of Amsterdam

(Accessed through GBIF Data Portal, MaNIS Data portal, HerpNET data portal, ORNIS data portal, and MaPSTeDI Map service between January and February 2007).
